# Laboratory Diagnostic Methods and Antibiotic Resistance Patterns of *Staphylococcus aureus* and *Escherichia coli* Strains: An Evolving Human Health Challenge

**DOI:** 10.3390/diagnostics12112645

**Published:** 2022-10-31

**Authors:** Feras Alzaben, Shawkat Fat’hi, Ayman Elbehiry, Maha Alsugair, Eman Marzouk, Adil Abalkhail, Abdulaziz M. Almuzaini, Mohammed Rawway, Mai Ibrahem, Wael Sindi, Turki Alshehri, Mohamed Hamada

**Affiliations:** 1 Department of Veterinary Medicine, College of Agriculture and Veterinary Medicine, Qassim University, Buraydah 52571, Saudi Arabia; 2 Department of Preventive Medicine, King Fahad Armed Forces Hospital, Jeddah 23311, Saudi Arabia; 3 Department of Food Hygiene, Faculty of Veterinary Medicine, Assiut University, Assiut 71515, Egypt; 4 Department of Public Health, College of Public Health and Health Informatics, Qassim University, Al Bukayriyah 52741, Saudi Arabia; 5 Department of Bacteriology, Mycology and Immunology, Faculty of Veterinary Medicine, University of Sadat City, Sadat City 32511, Egypt; 6 Biology Department, College of Science, Jouf University, Sakaka 42421, Saudi Arabia; 7 Botany and Microbiology Department, Faculty of Science, Al-Azhar University, Assiut 71524, Egypt; 8 Department of Public Health, College of Applied Medical Science, King Khalid University, Abha 61421, Saudi Arabia; 9 Department of Aquatic Animal Medicine and Management, Faculty of Veterinary Medicine, Cairo University, Cairo 12211, Egypt; 10 Dental Department, Alhada Armed Forces Hospital, Taif City 26792, Saudi Arabia; 11 Department of Food Hygiene & Control, Faculty of Veterinary Medicine, Sadat City University, Sadat City 32511, Egypt

**Keywords:** public health, antimicrobial susceptibility, peptide mass fingerprinting, *E. coli*, *S. aureus*, minced meat

## Abstract

Raw ground meat is known as a transmission vehicle for biological agents that may be harmful to human health. The objective of the present study was to assess microbiological quality of the ground meats. A total of 280 samples of local and imported chilled meats were randomly collected from retail shops in Buraydah City, Saudi Arabia. The meat samples were microbiologically analyzed using standard methods, peptide mass fingerprinting (PMF) technique, MicroScan Walkaway System (MicroScan) and qPCR System. The imported meat was more bacterially contaminated than local meat, with variable contamination degrees of *Staphylococcus aureus* (40.33%), *Escherichia coli* (36.13%), *Hafnia alvei* (7.56%), *Pseudomonas* spp. (6.72%), *Salmonella* spp. (5.88%) and *Aeromonas* spp. (3.36%). PMF verified all the isolated bacteria by 100%, compared to 75–95% achieved by MicroScan. The gene encoding flagellin (*fliC*) was recognized in 67.44% of *E. coli* strains, while the thermonuclease (*nuc*) and methicillin resistance (*mec*A) genes were detected in 100% *S. aureus* and 39.6% of methicillin-resistant *S. aureus* (MRSA) strains, respectively. The *S. aureus* and *E. coli* strains were highly resistant to multiple antibiotics (e.g., ampicillin, amoxicillin-clavulanic acid and cephalothin). For identifying various foodborne pathogens, PMF has been recognized as a powerful and precise analytical method. In light of the increasing use of PMF to detect multidrug-resistant bacteria, this study emphasizes the need for improved ways of treating and preventing pathogens, as well as setting up monitoring systems to guarantee hygiene and safety in meat production.

## 1. Introduction

Meat is a common carrier of foodborne diseases and hence linked to foodborne epidemics. Defective management practices may occur at any level of the farm-to-table process, increasing the risk of contamination and disease transmission in foods [1]. Contaminated food hazards principally depend on food handlers’ health conditions, as well as their personal hygiene, expertise and training. Consumption of microbial-infected foods or microbial toxins cause human diseases [2]. Foodborne illness is a global public health threat; however, most cases occur in developing countries due to insufficient food management procedures, lack of public awareness, lack or inadequacy of hygiene services, insufficient food safety legislation, ineffective regulatory disciplines and lack of financial resources [3].

Animal-derived foodborne pathogens, particularly from contaminated meat products, are widely considered to be the primary vectors of foodborne outbreaks. Food safety strategies, which include preparation, processing, packing, delivery, transit and storage, can be developed using data analysis of foodborne outbreaks [4,5]. Foodborne infection prevention and control necessitate appropriate food handler preparation, which includes preventing people with abscesses or further skin abrasions from touching foods, as well as appropriate food preparation, cooking and storage; food should be kept at 4 °C or lower temperatures to prevent bacterial growth and production of toxins [6]. It is important that offered beef products are safe, include low rates of spoilage and meet the stipulated appearance, composition, flavor, packaging and color specifications. Therefore, heavily microbial-contaminated items are undesirable [7].

Microbiological agents (enterococci, enterobacteria and fecal coliforms) are crucial for meat assessment, because they serve as markers of hygiene and microbial quality throughout manufacturing, handling and shelf-life prediction, as well as ensuring continuous cold chains [7]. *Staphylococcus* (*S.*) *aureus*, *Escherichia* (*E.*) *coli*, *Salmonella* spp., *Campylobacter* spp. and *Listeria monocytogenes* are the world’s most common foodborne pathogens. These pathogens and their toxins cause symptoms ranging from gastrointestinal problems to paralysis and death [5,8]. Antimicrobial resistance has been highlighted as one of the most serious hazards to public health and, therefore, one of the most pressing concerns of the 21st century [7]. Antibiotic treatment advancements have resulted in the emergence and enhancement of drug-resistant pathogens, which can cause a variety of problems, such as increased mortality and treatment costs, treatment failure, decreased efficiency of the infection control and spread of resistant pathogens to the community [9]. Antibiotic-resistant bacteria in foods include direct threats to customers, rather than the indirect risk of horizontal spread of bacterial resistance genes [7].

Meat contamination with resistant *S. aureus* and *E. coli* increases dramatically during slaughter process, indicating secondary contamination from the slaughterhouse environment [10]. Due to high levels of antimicrobial resistance, *E. coli* in processed meat products poses serious potential hazards to consumers. From the standpoint of public health, this worrying reality necessitates deployment of efficient methods to decrease levels of bacterial antibiotic resistance in these commodities [7]. Flagella encoded by the *fli*C gene affect bacterial virulence in multiple ways, including motility and adhesion to the host cells. The *fli*C gene helps bacterial movement and is critical for bacterial distribution in the host colon and other organs. Regarding public health, *E. coli* includes several antibiotic-resistant genes, which is a serious concern due to gene transfer risks to other bacteria [7,11].

The *S. aureus* has been identified as a zoonotic pathogen that cause food poisoning epidemics and serious illnesses in humans [12]. Staphylococci includes several virulence factors, including *nuc* gene that hydrolyzes DNA and RNA in the host cells, causes tissue destruction and microbial spread. Furthermore, the gene aids bacterial escape from neutrophils, allowing bacteria to avoid the host defense mechanisms. Because the *mec*A gene in *S. aureus* is responsible for β-lactam resistance, it includes health threats to people and animals within the food chain. Several studies have revealed that *S. aureus* strains are resistant to several antimicrobials [13]. Therefore, frequent monitoring of antibiotic resistance is not only necessary to collect evidence on the various aspects of this problem, but it is also necessary to prepare control procedures. The current study was designed to assess bacteriological statuses of local and imported ground meats and susceptibilities of the isolated *E. coli* and *S. aureus* to various antibiotics in Buraydah, Saudi Arabia.

## 2. Materials and Methods

### 2.1. Ethical Statement

This study did not require approval from ethics committees or written consent from participants, since it involved no human or animal participants. The bacterial strains came directly from the results of regular diagnostic tests. As a result, the investigation did not obtain samples from patients or animals.

### 2.2. Sampling, Preparation, and Bacterial Isolation

In total, 280 samples (300 g each) of chilled ground meats (140 samples of both beef and mutton meats; 70 samples of both local and imported meats) were collected from four retail markets in Buraydah City, Saudi Arabia, from December 2018 to November 2019. The samples were transferred into sterile plastic bags and stored under cold conditions for a maximum of 2 h, followed by isolation within this time. From each sample, 25 g were transferred to a clean blender jar and mixed with 225 mL of physiological saline solution (0.85% NaCl). Homogenization was carried out for 2 min at 1500–2000 rpm to provide a homogenate (1:10) that was used to prepare decimal serial dilutions [14]. For the recording of *S. aureus*, three decimal dilutions (10^−4^, 10^−5^ and 10^−6^) of each mixed sample were carried out. Then, 100 μL of each dilution were distributed on the surface of sterilized Barid-Parker agar plates (Sigma Aldrich, USA) in duplicates and incubated at 37 °C for 48 h. Suspicious colonies (black and glossy with thin white borders, surrounded by pure borders) were reported as *S. aureus*.

Moreover, *E. coli* strains were isolated on Eosin-Methylene-Blue (EMB) agar (Sigma Aldrich, USA). Briefly, 100 μL of the microbial dilution were cultured on EMB agar plates and incubated at 37 °C for 48 h. Colonies of E. coli exhibited a green metallic sheen when reflected light was present, with a dark or even black center when light was transmitted. Technically, *E. coli* DSM 498 and *S. aureus* ATCC 29,213 were used as positive controls in this study. Isolates were stored at −20 °C using Cryobank tubes for further studies.

### 2.3. Biochemical Identification of Staphylococcus aureus and Escherichia coli Using the Microscan WalkAway System

Using the MicroScan WalkAway device (Beckman Coulter, Brea, CA, USA), chemical analysis was carried out according to the method previously described by MacVane and Nolte [15]. For each panel, *S. aureus* ATCC 29,213 and *E. coli* ATCC 25,922 were used as reference strains [16].

### 2.4. Protein Analysis of Staphylococcus aureus and Escherichia coli Isolates Using Peptide Mass Fingerprinting

The protocol used for the proteomic identification of various isolates of *S. aureus* and *E. coli* from chilled ground meat samples was based on the method described by Bruker Daltonics, Bremen, Germany, using ethanol-formic acid-acetonitrile extraction protocol [17].

#### 2.4.1. Preparation of α-cyano-4-hydroxycinnamic Acid Matrix Solution

Briefly, 250 µL of the standard solvent solution were added to a tube of α-cyano-4-hydroxycinnamic acid (HCCA). Then, HCCA was dissolved by mixing thoroughly at room temperature until the solution became clear [18].

#### 2.4.2. Preparation of Bacterial Test Standard

First, in vitro diagnostic bacterial test standard (BTS) pellet was inoculated with 50 μL of the standard solvent solution and carefully mixed at 25 °C. Centrifugation of the mixture solution was carried out at 13,000 rpm for 2 min (Eppendorf Model 5430, Hamburg, Germany). Then, 5 μL of the supernatant were transferred into a microtube and stored at −20 °C for further use [19].

#### 2.4.3. Ethanol-Formic Acid-Acetonitrile Extraction Protocol

Briefly, 300 µL of purified water was transferred into a sterile microtube. Then, a single fresh colony was inoculated onto the microtube and mixed. Absolute ethanol (900 µL) was added to the microtube, diversified lengthily, and centrifuged at 5.008–5.871× g for 2 min (Eppendorf Model 5430, Hamburg, Germany). The supernatant was discarded, and centrifugation was carried out and all the residual ethanol was removed by careful pipetting. Absolute ethanol-pellet was dried at 25–27 °C for 2–3 min. Then, 80 µL of formic acid (70%) were added to the pellet and mixed well by pipetting and/or vortexing. Then, 80 µL of pure acetonitrile (70%) were added to the mixture and mixed. Then, centrifugation was carried out for 2 min at maximum speed, and 1 µL of the supernatant was inoculated onto a MALDI target plate and set to dry at 23–25 °C. Then, 1 µL of HCCA solution was overlaid onto the entire spot within 1 h and set to dry at 23–25 °C. Running of the samples was carried out using Compass and Flex-Control Software of the Microflex LT Device [18].

#### 2.4.4. Data Analysis in Peptide Mass Fingerprinting

Assessment of the score value for unknown spectra from 0.00 to 3.00 was carried out by comparison between the unidentified spectra with those stockpiled in the database of Bruker Software. Measures of Bruker Diatonic were used for the precision of the isolate discovery. Device was operated appropriately once the score value reached 3.0 from 2.3; moreover, identification of various microbes at species and genus levels were carried out exactly at score values ranging 2.00–2.29 and 1.700–1.999, respectively. Moreover, identification was not dependable when the score varied from zero to 1.69. Varied spectra produced by the PMF Software were recorded in m/z ranging 2000–18,000 Da. Based on the principal component analysis (PCA), various bacterial species were differentiated, and results verified in a three-dimensional (3-D) PCA, which was generated by the Bruker Software [20].

### 2.5. Molecular Analysis of Staphylococcus aureus and Escherichia coli Strains Using RT-PCR DNA Extraction

In this study, DNA was extracted from the identified *S. aureus* and *E. coli* strains based on the manufacturer’s instructions, using the semi-automated QuickGene-810 Device (AutoGen, Japan) and Quick Gene DNA Tissue Kit S (DT-S) (Kurabo Industries Ltd., Japan). Then, DNA quality was assessed using NanoDrop 2000 Spectrophotometer (Fisher Scientific, USA) [21]. The strains were recognized for their virulence by *nuc* and *mec*A genes for identification of *S. aureus* and *fli*C gene for *E. coli*. A specific 16S rRNA region was prudently selected for identifying the bacteria [21]. SYBR Green qPCR for *S. aureus* and *E. coli* strains was carried out using qPCR system. The primer sequences were as follows: *nuc* forward primer, nuc1, 5’GCG ATT GAT GGT GAT ACGGTT3′ and reverse primer, nuc2, 5′AGC CAA GCC TTG ACG AAC TAA AGC3′ [22]; mecA forward primer, mecA1, 5′ GCA ATC GCT AAA GAACTA AG 3′ and reverse primer, mecA2, 5′ GGG ACC AAC ATA ACC TAA TA 3′ [22]; and *fliC* forward primer, *fliC*1, 5′GGG ACC AAC ATA ACC TAA TA 3′ and reverse primer *fliC*2, 5′ GACTCCATCCAGGACGAAA 3′ [23], were used in the current study. In brief, 20-µL reaction volume was used, including 10 µL of master mix (2×) (Applied Biosystem, Waltham, MA, USA), 1 µL of each forward and reverse primers, 1 µL of the template DNA and 7 µL of purified water. All reactions were carried out twice. Amplification was carried out as follows: initial denaturation at 50 °C for 2 min and 95 °C for 2 min; then 40 cycles, each included 95 °C for 15 s and extension at 60 °C for 1 min. Intensification findings were described by plotting Delta Rn (ΔRn) [21].

### 2.6. Antimicrobial Susceptibility of Staphylococcus aureus and Escherichia coli Isolates Using Microscan

Based on the method described by Baker et al. [24] and protocols by Siemens Healthcare Diagnostics, USA, the MicroScan WalkAway Diagnostic System detected degrees of susceptibility of 48 *S. aureus* and 43 *E. coli* strains against 14 antibiotics (as shown in Table 1). Agar diffusion (Kirby-Bauer) was used as a reference method to confirm MicroScan assay MIC results [25]. In brief, the antibiotic panel was firstly removed from its foil; the barcode label was applied. Sensititre Nephelometer (TREK Diagnostic Systems, Ashford, Kent, UK) was used for the adjustment of bacterial turbidity using salt solution to achieve turbidity equivalent ca 1 × 108 CFU ml-1 after comparing the solution with 0.5 McFarland standard. Reference and MicroScan methods used the same adjusted inoculum. The inoculation wand pick was used to take the adjusted inoculum. After inserting the wand into Rapid Inoculation System-D (Diluents), we thoroughly shook it to dislodge all its contents into the diluents. A cover was placed on top of the Renok Disposable-D inoculator sets (plate) after all the contents had been carefully poured into the plate. Upon placing the Microscan Renok on top of the inoculator sets’ cover, fluid was aspirated into the Renok. As soon as the Renok was left on the panel, the fluid was discharged into it, and the panel’s cover was placed over it. Incubation was carried out on the panel using the Microscan Walkaway for 18 to 24 h, and the results were then interpreted using the Lab Pro LCD.

## 3. Results

### 3.1. Prevalence of Staphylococcus aureus and Escherichia coli in Beef and Mutton Chilled Ground Meat

From the 280 samples of chilled ground meat involved in our study (Table 2), 119 isolates were recovered from pure cultures. These isolates were known as follows: 40.33% *S. aureus*, 36.13% *E. coli*, 7.56% *Hafnia alvei*, 6.72% *Pseudomonas* spp., 5.88% *Salmonella* spp. and 3.36% *Aeromonas* spp. Based on our findings, *S. aureus* and *E. coli* were the major isolated bacterial isolates from both beef and mutton meat. Because of long-term transportation, an increased prevalence of bacteria was found in the imported meat than in the local meat, such as beef or mutton.

### 3.2. Biochemical Analysis of Staphylococcus aureus and Escherichia coli Isolates Using Microscan

Biochemical analysis of bacterial isolates was accomplished with the MicroScan WalkAway diagnostic system, compared with the PMF technique. One hundred and ten out of 119 (92.43%) microbial species were properly recognized as 95.83% (46/48) *S. aureus*, 93.02% (40/43) *E. coli*, 66.67% (6/9) *Hafnia alvei*, 75% (6/8) *Pseudomonas *sp., 85.71% (6/7) *Salmonella* sp. and 100% (4/4) *Aeromonas *sp.

### 3.3. Proteomic Identification of Staphylococcus aureus and Escherichia coli, and Other Microorganisms

The isolated bacteria were examined by PMF, and the resulted spectra were paralleled with the deposited spectra in the database of Microflex LT. A typical exploration of 48 *S. aureus* recovered from beef and mutton samples were illustrated by Compass software of Microflex LT. In the current analysis, PMF was able to identify 100% of isolated bacteria, with a log score fluctuating from 2.300 to 3.000 for eight strains of *S. aureus*, 11 *E. coli*, four *Hafnia alvei*, five *Pseudomonas*, three *Salmonella* sp. and three *Aeromonas *sp. Moreover, the log score was ranged from 2.00 to 2.29 for 40 strains of *S. aureus*, 32 *E. coli*, five *Hafnia alvei*, three *Pseudomonas*, four *Salmonella *sp. and one *Aeromonas *sp.

**Figure 1:**
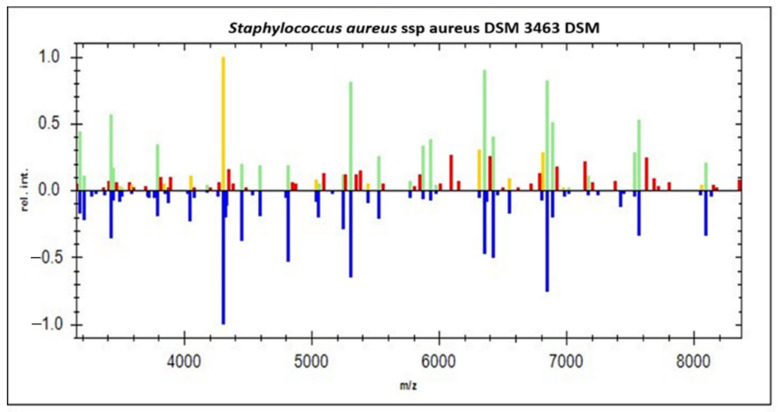
Comparison of the spectral protein profiles of 48 *Staphylococcus aureus* recovered from chilled ground meat samples with those of *Staphylococcus aureus* subspecies. *aureus* DSM 3463 as a reference strain using Compass Satellite Software. Blue color in the lower part of the spectra shows the deposited spectra used for matching patterns; green color in the upper part of the spectra shows excellent matched peaks, whereas red and yellow colors show mismatched and intermediate peaks, respectively.

Twenty protuberant ion peaks were distinguished in the original bands from the zone, extended from 3000 to 16,000 Daltons (Da), and strong signals were demonstrated between 3785 and 7000 Da. As can be seen in (Figure 1), all identified strains of *S. aureus* were coordinated with eight reference isolates of *S. aureus* stored in the Compass software library (*S. aureus* ATCC 29213, *S. aureus* DSM 799, *S. aureus* DSM 20232, *S. aureus* ATCC 25923, *S. aureus* ATCC 33591, *S. aureus* DSM 346, *S. aureus ssp. aureus* DSM 20231, *S. aureus ssp. aureus* DSM 3463). Likewise, several spectral proteins were scattered for most *E. coli* strains in the original bands from the zone, ranging from 3000 to 13,500 Da with a few strains scattered until 18,000 Da, and higher peak signals were observed between 3000 and 10,000 Da.

**Figure 2:**
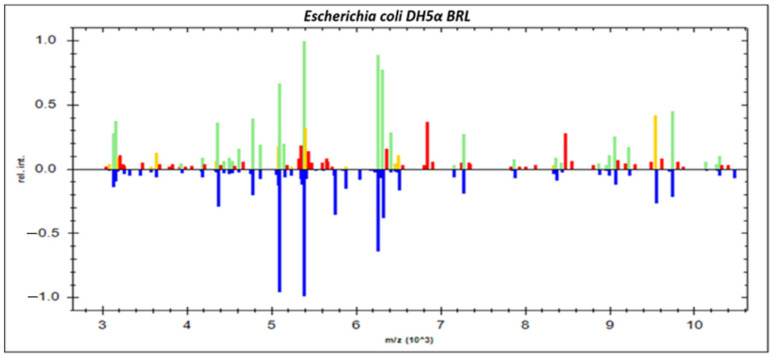
Comparison of the spectral protein profiles of 43 *Escherichia coli* strains recovered from chilled ground meat samples with those of *Escherichia coli* DH5α as a reference strain using Compass Satellite Software.

As shown in Figure 2, all recognized *E. coli* strains were coordinated with four reference *E. coli* strains deposited in the Compass software library (*E. coli* ATCC 25922, *E. coli* DH5α, *E. coli* DSM 1576, *E. coli* RV412). A real gel image was created by the Compass software programmed in Microflex LT device for illustration of protein profile for 48 recognized *S. aureus* strains and 43 *E. coli* strains. The spectral protein of *S. aureus* strains was scattered in the range from 3.000 Da to 10.000 Da, with strong peaks demonstrated between 3.700 and 7.000 Da. Likewise, the gel view of protein for *E. coli* strains was scattered in the range from 3.000 Da to 13.500 Da, with higher peaks demonstrated between 3.000 and 10.000 Da.

Our findings indicated that the gel views of protein for *S. aureus* and *E. coli* strains recovered from both beef and mutton meat samples were matched with the spectral protein profiles (single peak intensities) for both bacteria. The principal component analysis (PCA) represents a supplementary calculated tool extracted from Compass software (Version 3.1.14) of the Microflex LT device for analyzing datasets to illustrate the degree of resemblance and variety of various spectra of protein profile. Additionally, the PCA was used for grouping the isolates, as stated by the different algebraic assessments. Several spectral proteins for *S. aureus* and *E. coli* strains were established in 3-D PCA. Every spectrum was stated via dot, and the various colors demonstrate the reflected group contribution, in which every dot represented by one spectrum of the protein side view.

### 3.4. Molecular Identification of Staphylococcus aureus and Escherichia coli by SYBR Green Real-Time PCR

**Figure 3:**
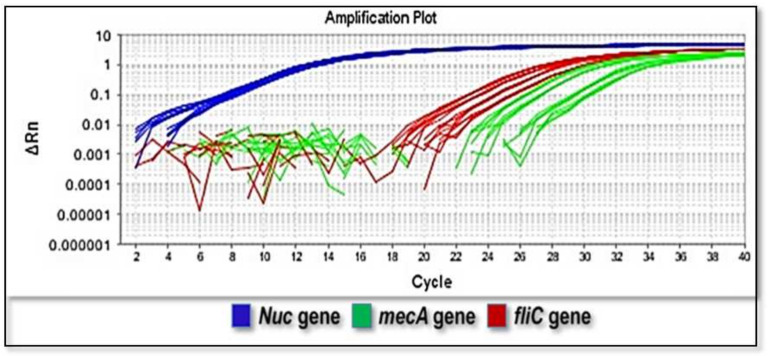
SYBR Green RT-PCR amplification plot for the identification of *nuc* (blue color) and *mec*A (green color) genes in *Staphylococcus aureus* and *fli*C gene (red color) in *Escherichia coli* strains.

Real-time PCR was then accomplished to approve the PMF findings. The oligonucleotide primers for the *S. aureus* and *E. coli*, *nuc* and *mecA* and *fliC* genes were applied to recognize the pathogenicity of *S. aureus* and *E. coli* strains. The magnification with these primers generated amplicons of the anticipated molecular weights. Each gene was amplified independently. According to our findings, the *nuc* gene (100%) was detected in all *S. aureus* strains under investigation, whereas the *mec*A gene was detected in 39.6% (19/48) of *S. aureus* strains. The *fliC* gene was recognized in 29 out of 43 (67.44%) of *E. coli* strains (Figure 3).

### 3.5. Antimicrobial Resistance of Identified Staphylococcus aureus and Escherichia coli Strains

MicroScan was applied to test the degree of *S. aureus* and *E. coli* resistance to numerous antibiotics frequently utilized for the management of both pathogens. As revealed in Table 3, all *S. aureus* strains (100%) were resistant to beta-lactam penicillins (ampicillin), beta-lactam/beta-lactam inhibitors (amoxicillin-clavulanic acid), first-generation cephalosporins (cephalothin) and quinolone (nalidixic acid) (Table 3). A total of 87.5%, 39.6%, 39.6%, 31.2%, 22.9% and 12.5% of *S. aureus* isolates were resistant to fourth-generation cephalosporins (cefepime), beta-lactam penicillins (piperacillin), second-generation cephalosporins (cefoxitin), norfloxacin (fluoroquinolone), ciprofloxacin (fluoroquinolone) and tetracyclines (tetracycline), respectively.

In contrast, the sensitivity of *S. aureus* was 100% for carbapenem (imipenem), 58.3% for Sulfonamides (Cotrimoxazole) and 41.7% for Nitrobenzenes (Chloramphenicol). Likewise, regarding the *E. coli* strains, the percentage of resistant was 100% for beta-lactam penicillins (ampicillin), beta-lactam/beta-lactam inhibitors (amoxicillin-clavulanic acid), first-generation cephalosporins (cephalothin) and second-generation cephalosporins (cefoxitin) (Table 3). In contrast, all strains of *E. coli* showed strong activity (100%) against sulfonamides (cotrimoxazole) and carbapenem (imipenem). Moreover, 58.1% and 53.5% of *E. coli* strains were susceptible to nitrobenzenes (chloramphicol) and Quinolone (nalidixic acid), respectively.

## 4. Discussion

Bacterial contamination of chilled ground meat can occur during any stage of meat production and processing, from the point of slaughter until reaching the consumer, and even during its preparation in the kitchen. In veterinary practice, the inaccurate usage of antibiotics may lead to bacterial antimicrobial resistance, which constitutes a threat to human health, so bacteria and their resistance to the commonly used antibiotics must be identified [26]. Our results showed that the occurrence of *S. aureus* and *E. coli* strains isolated from chilled ground meat was greater than other bacterial pathogens, and the imported ground meat was more contaminated than the local samples. *S. aureus* (40.33%) and *E. coli* (36.13%) were harbored on the tested chilled ground meat samples. Unhygienic meat-handling practices were clearly demonstrated by this pattern of findings. Therefore, the actual cause of meat spoilage by *S. aureus* and *E. coli* may be the personal unhygienic activities of workers during the management and treating of minced red meat [26].

Several studies have recorded variable incidence levels, which could be due to variations in meat sample type, handling practices and their geographic location. In contrast with other research, it was established that the incidence rate of *S. aureus* and *E. coli* in retail meat marketplaces is widely varied in different countries [27]. Similarly, to our recorded results, around 37% of meat samples exhibited the existence of coagulase-positive *S. aureus* [28]. Other studies showed that 29.4% of meat samples carried *S. aureus*. Additionally, other researchers have reported cross-contamination of meat samples through contact areas in butcher’s shops by *S. aureus* [29]. In a comparable study, *E. coli* strains have been found at 43.75% and 29.17% in minced meat and contact surfaces of butcher’s shops, respectively [28]. In other research, *E. coli* strains were found at a prevalence of 30% and 32% in minced meat and contact surfaces at butcher’s shops [30].

Our findings, as well as those of others, emphasize the need for good hygiene at retail stores and during transportation and handling, to reduce the risk of bacterial spread from meat products to humans. Mass spectrometry technology is thought to be a good means of microbial detection, due to its rapidity, accuracy, precision, easiness, reproducibility and low-priced materials. It provides a pronounced chance for recognition of different pathogens that are hard to detect via biochemical techniques [31]. From this perspective, mass spectrometry technology provides a good opportunity for resolving the problem of recognition of microorganisms recovered from various foodstuffs. PMF represents a powerful and alternative practice for the principal detection of bacterial threats that might contaminate foodstuffs [31].

The bacterial strains isolated in this study included *S. aureus*, *E. coli*, *Hafnia alvei*, *Pseudomonas *sp., *Salmonella enterica* and *Aeromonas *sp. All these bacterial isolates were suitably recognized at a 100% rate via PMF species levels, with a log value greater than or equal to 2.00, while 92.43% of the same bacterial isolates were properly recognized by the Microscan. The findings of the present investigation were parallel with several prior studies using PMF as a validation instrument for the recognition of foodborne bacteria. PMF recognized a huge quantity of bacteria causing deteriorations of foodstuffs, such as *Escherichia*, *Salmonella*, *Staphylococcus* and *Pseudomonas* [32,33]. Applying PMF is a respected method for accurate detection of fastidious microorganisms, with a log rate greater than or equal to 2.00. Moreover, they exhibited that all bacterial isolates (100%) under examination were appropriately recognized, and the PMF seemed precise, lucrative and quick. Proof of identity of numerous microorganisms via PMF offers one of the best accurate techniques for microbial identification.

The mechanism of this technology depends mostly on the variations of spectra generated by field isolate of bacteria matched with the spectra deposited in the dataset [34]. Assessment of two PMF instruments (Microflex LT and VITEK MS) illustrated that the Microflex LT (Bruker Daltonics, Germany) is more accurate than Vitek Mass Spectrometry (BioMerieux, France) for microbial identification [35]. In the current study, the performance of PMF using Microflex LT for grouping of 119 bacterial isolates were assessed as typing technique. The findings of the MSP dendrogram demonstrated that most recognized microorganisms were closely associated with 14 *S. aureus* strains and nine *E. coli* strains reference strains deposited in the Bruker catalogue. MSP dendrogram represents a consistent method for demonstrating the capability of Microflex LT to display the degree of similarities and alterations between different kinds of bacteria [34].

All microbial isolates isolated in the present investigation were recognized by the Microflex LT device in approximately 1.5 h, while the detection of these bacteria by the Microscan can take several hours or days [36]. In the current investigation, when we compared the Microflex LT with the Microscan for repetitive detection of different microorganisms recovered from foodstuffs, the Microflex LT showed more dependable, fast and precise results than the Microscan. During this investigation, we confirmed that the Microflex LT was not only a rapid, cheap and specific tool for recognition of meat samples, but also for the grouping of various types of bacteria.

The cost of the Microflex LT device is considered to be one of the potential disadvantages. Nonetheless, considering the particularly inexpensive price of consumables and the opportunity of decreasing manual labour prices, the total cost/sample may be reduced over time compared with other methods. PMF is considered one of the best methodologies, because it depends mostly on the resemblances and dissimilarities in definite biomarkers. Not only is comparison with reference databases obligatory for attaining excellent identification results, but the quality of the reference information and the algorithm for recognition are also of high significance [36]. The algorithm for computerized discovery of diverse microbial isolates in the Microflex LT was established as a powerful and consistent recognition method. A partial amount of great strength peaks, frequently because of cytoplasmic proteins, have been selected as biomarkers [36].

Furthermore, the SYBR Green RT-PCR used in our investigation was successfully applied to approve the PMF findings. The oligonucleotide primers exactly targeting regions of the *S. aureus* and *E. coli*, *nuc* and *mec*A and *fliC* genes were utilized to recognize the pathogenicity of *S. aureus* and *E. coli* strains. The magnification of these primers generated amplicons of the predictable molecular weights. Every gene was amplified distinctly. According to our results, the nuc gene (100%) displayed in all *S. aureus* strains under investigation, whereas the *mec*A gene displayed in 39.6% of *S. aureus* strains. The *fliC* gene was recognized in 67.44% of *E. coli* strains. The growing usage of antibacterial drugs in livestock production and human medicine is considered a critical aspect in the development of the bacterial resistance to various antibiotics [37].

The MicroScan was applied to test the degree of *S. aureus* and *E. coli* resistance to various antibacterial drugs that are frequently used for treatment of both pathogens. The results achieved exhibited that all *S. aureus* strains (100%) were resistant to ampicillin, amoxicillin-clavulanic acid, first-generation cephalosporins (cephalothin) and quinolone (nalidixic acid). A total of 87.5%, 39.6%, 39.6%, 31.2%, 22.9% and 12.5% of *S. aureus* isolates were resistant to fourth-generation cephalosporins (cefepime), beta-lactam penicillins (piperacillin), second-generation cephalosporins (cefoxitin), norfloxacin (fluoroquinolone), ciprofloxacin (fluoroquinolone) and tetracyclines (tetracycline), respectively. In contrast, the sensitivity of *S. aureus* was 100% for carbapenem (imipenem), 58.3% for Sulfonamides (Cotrimoxazole) and 41.7% for Nitrobenzenes (Chloramphenicol).

However, another study has pointed out that the antimicrobial resistance profile of *S. aureus* from meat demonstrated resistance against penicillin (90.97%), ciprofloxacin (61.80%) and tetracycline (45.14%). Furthermore, low resistance levels, varying from 2 to 9%, were detected for chloramphenicol, clindamycin, ceftriaxone and oxacillin [38]. A comparatively high incidence of *S. aureus* and high levels of antibiotic resistance was found among the isolates from ground meat, thereby suggesting the prospective role of meat in the distribution of methicillin-resistant *S. aureus* (MRSA) among customers. Antibacterial-resistant *S. aureus*, particularly MRSA, is a concern, due to its ability to generatedifficult-to-treat, serious infections [27]. Contamination of meat products, meat industry workers, mainly raw meat and retail markets has been achieved by MRSA strains. The genomic system was accountable for the advancement of methicillin/oxacillin resistance via the asset and inclusion of staphylococcal chromosome cassette *mec* (SCCmec) elements, which carry antibiotic resistance factors [38].

Furthermore, the obtained results showed that the resistance rates for the b-lactam antibiotics, including ampicillin (100%) and amoxicillin (100%), are close to the percentage (90.97%) and (92.3%) of strains resistant to penicillin formerly stated by Zehra et al. [27]. All mecA positive isolates were tolerant if penicillin and cefoxitin, which was in accordance with the results of Heikinheimo et al. [39]. On the other hand, there were variable ratios of antimicrobial resistance of *S. aureus* to ampicillin (70.1%) and tetracycline (47.3%), in addition to other antimicrobial resistance of *S. aureus* isolates to cefoxitin (22.8%) gentamicin (12.2%) and chloramphenicol (5.2%) [40]. Therefore, there is a possible correlation between the overuse or misuse of antibiotics in veterinary practice and the emergence of resistant human pathogens.

Concerning *E. coli*, antibiotic resistance percentage was 100% for beta-lactam penicillins (ampicillin), beta-lactam/beta-lactam inhibitors (amoxicillin-clavulanic acid), first-generation cephalosporins (cephalothin) and second-generation cephalosporins (cefoxitin). In contrast, all strains of *E. coli* strongly showed activity (100%) against sulfonamides (cotrimoxazole) and carbapenem (imipenem). Moreover, 58.1% and 53.5% of *E. coli* strains were susceptible to nitrobenzenes (chloramphenicol) and Quinolone (nalidixic acid), respectively. A similar study examined the antimicrobial resistance of *E. coli* strains recovered from various food samples, and indicated that the percentage of resistance against tetracycline, and sulphamethoxazole trimethoprim was 41.35% and 19.55%, respectively, whereas lower resistance was detected against gentamicin, and nitrofurantoin [38].

In another investigation, the proportion of *E. coli* resistance isolates isolated from food of animal source against tetracycline, ampicillin and trimethoprim/sulfamethoxazole were 57%, 38% and 37%, respectively [37]. Similarly, in different countries (e.g., U.S.A., U.K., Canada, Spain and Germany), *E. coli* isolated from retail meat and poultry samples were highly resistant to tetracycline due to the excessive application of tetracycline by-products [37]. *E. coli* has been reported to be commonly used as a marker of antibiotic resistance of bacteria within the community [3]. Most of the obtained *E. coli* isolates were resistant to ampicillin, amoxicillin and cephalothin (100%), alongside tetracycline (30.2%) and to Chloramphenicol (18.6%), which is analogous to results reported by Martínez-Vázquez et al. [41], who found that the percentage of resistance to cefazolin, ampicillin and tetracycline were 91.8%, 90.5% and 67.7%, respectively.

Furthermore, there were similar results revealed: 100% of *E. coli* isolates were resistant to penicillin and existed varying ratios of antibiotic resistance to chloramphenicol (64.29%), to ampicillin (57.14%) and to tetracycline (46.43%) [42]. Likewise, ampicillin resistance has been reported to be 96.7% in healthy cattle in Nigeria by Ogunleye et al. [43]. Wholesale meats can hold antimicrobial-resistant *E. coli* isolates, including those resistant to clinically important antibiotics, and these might be a major resistance reservoir [3]. Due to the habitual application of antibiotics in animal feed, meat is often tainted with antibiotic-resistant *E. coli*. Microorganisms from the animal that resist various kinds of antibiotics have a harmful effect when used to treat human diseases (e.g., aminoglycosides, fluoroquinolones and third- and fourth-generation cephalosporins) [37].

In conclusion, the bacteriological status of chilled ground meat included different types of bacteria, and the commonly isolated bacteria were *S. aureus* and *E. coli*. The use of PMF was largely able to identify the different microbes isolated from meat of different animal origin. Furthermore, the degree of contamination was higher in imported ground meat than local. The isolated *S. aureus* and *E. coli* strains exerted a higher degree of resistance for more than three antimicrobial agents. To guide future study, a full understanding of the bacterial limits and the heterologous patterns of antibiotic susceptibility/resistance is required. As a food safety issue that occurs frequently in the food chain, it is critical to address antibacterial resistance incidents.

## Figures and Tables

**Table 1:** Zone Diameter Interpretative references (NCCLS *) for various antimicrobials against *Staphylococcus aureus* and *Escherichia coli* strains isolated from chilled ground meat samples.

Antimicrobial Drug	Conc. (µg)	*S. aureus* Inhibition Zone (mm)			*E. coli* Inhibition Zone(mm)		
		S	I	R	S	I	R
Ampicillin	10	≥36	27–35	≤26	≥15	16–22	≤15
Amoxicillin-clavulanic acid	20	≥37	28–36	≤27	≥25	18–24	≤17
Gentamicin	10	≥28	19–27	≤17	≥27	19–26	≤18
Cefoxitin	30	≥30	23–29	≤22	≥30	23–29	≤22
Cephalothin	30	≥38	29–37	≤28	≥22	15–21	≤14
Trimethoprim/sulfamethoxazole	25	≥19	16–18	≤15	≥16	11–15	≤10
Nalidixic Acid	30	≥29	22–28	≤21	≥29	22–28	≤21
Norflaxcin	10	≥29	17–28	≤16	≥36	28–35	≤27
Amikacin	30	≥27	20–26	≤19	≥27	19–26	≤18
Cefepime	30	≥30	23–29	≤22	≥38	31–37	≤30
Ciprofloxacin	5	≥31	22–30	≤21	≥41	30–40	≤29
Chloramphenicol	30	≥18	13–17	≤12	≥18	13–17	≤12
Tetracycline	30	≥31	24–30	≤23	≥26	18–25	≤17
Piperacillin	100	≥31	24–30	≤23	≥31	24–30	≤23
Imipenem	10	≥ 16	14–15	≤13	≥16	14–15	≤13

* National Committee for Clinical Laboratory Standards, *S. aureus* = *Staphylococcus aureus, E. coli = Escherichia coli.*

**Table 2:** Proportions of the bacteria isolated from local and imported chilled ground meat samples.

Total Isolates	Type of Meat				Type of Bacteria
	Mutton		Beef		
	Imported	Local	Imported	Local	
48	20	7	13	8	*Staphylococcus aureus*
43	21	3	15	4	*Escherichia coli*
9	2	0	7	0	*Hafnia alvei*
8	2	1	3	2	*Pseudomonas *sp.
7	3	1	2	1	*Salmonella *sp.
4	2	1	1	0	*Aeromonas *sp.
119	50	13	41	15	*Total number*

**Table 3:** Assessment of 14 antimicrobial agents against 48 *Staphylococcus aureus* and 43 *Escherichia coli* strains recovered from chilled ground meat samples.

Drug Class	*S. aureus*			*E. coli*		
	Resistant	Intermediate	Sensitive	Resistant	Intermediate	Sensitive
Β-lactam penicillins						
Ampicillin	100	0	0	100	0	0
Piperacillin	39.6	61.4	0	0	58.1	42
Β-lactam/β-lactam inhibitors						
Amoxicillin-clavulanic acid	100	0	0	100	0	0
Aminoglycoside						
Gentamicin	0	100	0	0	100	0
Amikacin	0	100	0	0	65.1	35
Cephalosporins 1st						
Cephalothin	100	0	0	100	0	0
Cephalosporins 2nd						
Cefoxitin	39.6	0	60.4	100	0	0
Cephalosporins 4th						
Cefepime	87.5	0	12.5	14	86	0
Sulfonamides						
Cotrimoxazole	0	41.7	58.3	0	0	100
Quinolone						
Nalidixic acid	100	0	0	0	46.5	53.5
Fluoroquinolone						
Norfloxacin	31.2	69.8	0	0	95.34	4.7
Ciprofloxacin	22.9	70.8	6.3	0	100	0
Nitrobenzenes						
Chloramphenicol	0	58.3	41.7	18.6	23.3	58
Tetracyclines						
Tetracycline	12.5	83.3	4.2	30.2	55.8	14
Carbapenem						
Imipenem	0	0	100	0	0	100

## Data Availability

Not applicable.
